# Guillain-Barré Syndrome Following Influenza Vaccination: Diagnostic Challenges in an Uncommon Presentation

**DOI:** 10.7759/cureus.113736

**Published:** 2026-07-31

**Authors:** Ita A Sanchez, Victor A Capistran, Miguel A Luna, Asher I Licona

**Affiliations:** 1 Internal Medicine, General Hospital Xoco, Mexico City, MEX; 2 Internal Medicine, Hospital General Regional de Orizaba No. 1 "Lic. Ignacio García Téllez", Orizaba, MEX; 3 Medicine, Escuela Nacional de Medicina y Homeopatia, Instituto Politécnico Nacional, Mexico City, MEX

**Keywords:** autoimmune, demyelinating, demyelinating polyneuropathy, neurology, neuropathy

## Abstract

Guillain-Barré syndrome (GBS) is an acute, immune-mediated inflammatory polyradiculoneuropathy. It is currently recognized as the most common cause of acute flaccid paralysis worldwide. Bilateral facial palsy with paresthesias is a well-recognized regional variant of GBS, characterized by prominent bilateral facial weakness, distal extremities paresthesias, diminished tendon reflexes, and the absence of ophthalmoplegia, ataxia, or limb weakness.

We present a case of a 71-year-old male with recent flu vaccination who developed bilateral facial diplegia within 24 h, accompanied by paresthesias affecting the distal extremities with patchy hypoesthesia distributed over the lower limbs. The diagnosis of GBS was established following thorough clinical evaluation, neurological examination, and laboratory findings. This case may contribute to the timely recognition of this specific variant of GBS.

## Introduction

Guillain-Barré syndrome is a heterogeneous, acute, immune-mediated inflammatory polyradiculoneuropathy. Although reported globally, this disease remains rare, with an estimated incidence ranging from 0.81 to 1.91 cases per 100,000 person-years across Europe and North America. There is a male predominance, and the incidence increases with advancing age [1].

The disease’s clinical course has the following four stages: the first is a prodromal phase characterized by the presence of an infectious or non-infectious event that causes loss of immune tolerance. The second phase has progressive neuropathic symptoms that last less than four weeks. The plateau phase may last days, weeks, or even months, with an average of one to four weeks, to finally reach the recovery phase where the patient regains the ability to walk independently within six months of disease onset, with or without proper treatment [1].

Guillain-Barré syndrome (GBS) is heterogeneous, with multiple clinical variants. Bilateral facial weakness with paresthesias is an under-recognized and often underdiagnosed variant, with an incidence of one case per 5,000,000 individuals [2]. Clinically, it is manifested with facial diplegia associated with paresthesias in the distal extremities and hyporeflexia, without ophthalmoplegia, ataxia, or limb weakness [2].

## Case presentation

A 71-year-old male with hypertension and chronic coronary syndrome, who had received seasonal flu vaccination three weeks prior, presented to medical care due to unilateral nasolabial fold flattening and absence of forehead expression lines in the ipsilateral region, which progressed over 24 h to involve the contralateral facial musculature, along with a 72-h history of bulbar dysarthria and patchy paresthesias/hypoesthesia in the lower limbs without a defined dermatomal distribution.

Prior to hospital admission, the patient had no proximal or distal motor compromise, dysphagia, or diplopia. Also, he presented with an episode of diarrhea a week before the symptoms started, characterized by watery consistency, without mucus, blood, or tenesmus, with a frequency of three to five daily episodes and an approximate duration of three days; he self-medicated with two doses of trimethoprim/sulfamethoxazole, with spontaneous remission.

During the initial clinical assessment, the patient presented with normal vital signs, evidence of facial diplegia, bilateral masseter muscle weakness, impaired soft palate elevation, paresthesias, and patchy hypoesthesia in the lower limbs. There was no weakness or alteration in muscle tone in the upper or lower limbs. Muscle strength was formally assessed using the Medical Research Council (MRC) scale, with no evidence of weakness; gait was assessed and found to be unremarkable; and deep tendon reflexes were globally preserved.

**Figure 1:**
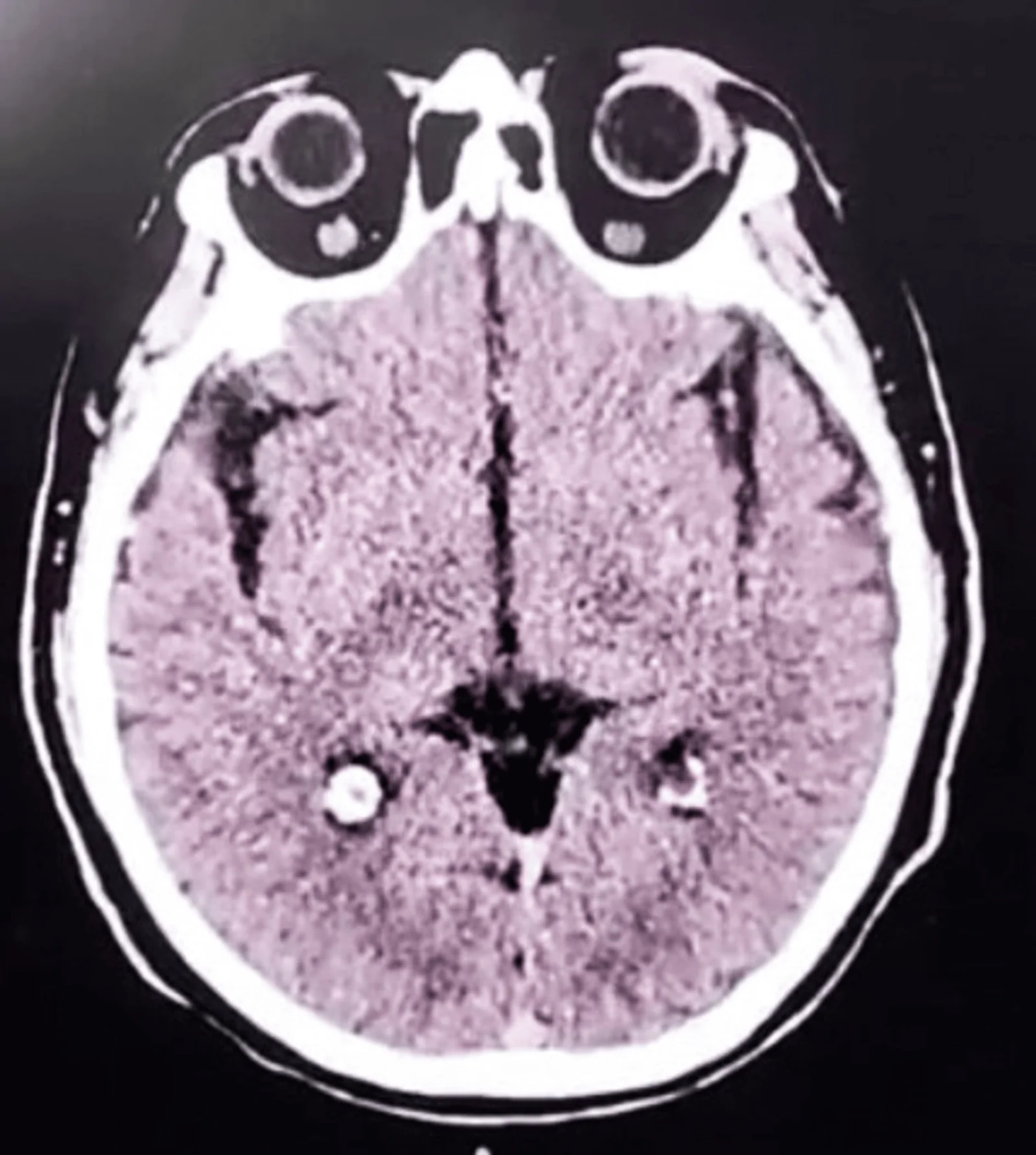
Axial non-contrast CT of the head at the level of the orbits and suprasellar cistern. CT: computed tomography, demonstrating no evidence of acute hemorrhage, mass effect, or other intracranial lesion. Ventricles and sulci are within normal limits for age.

**Figure 2:**
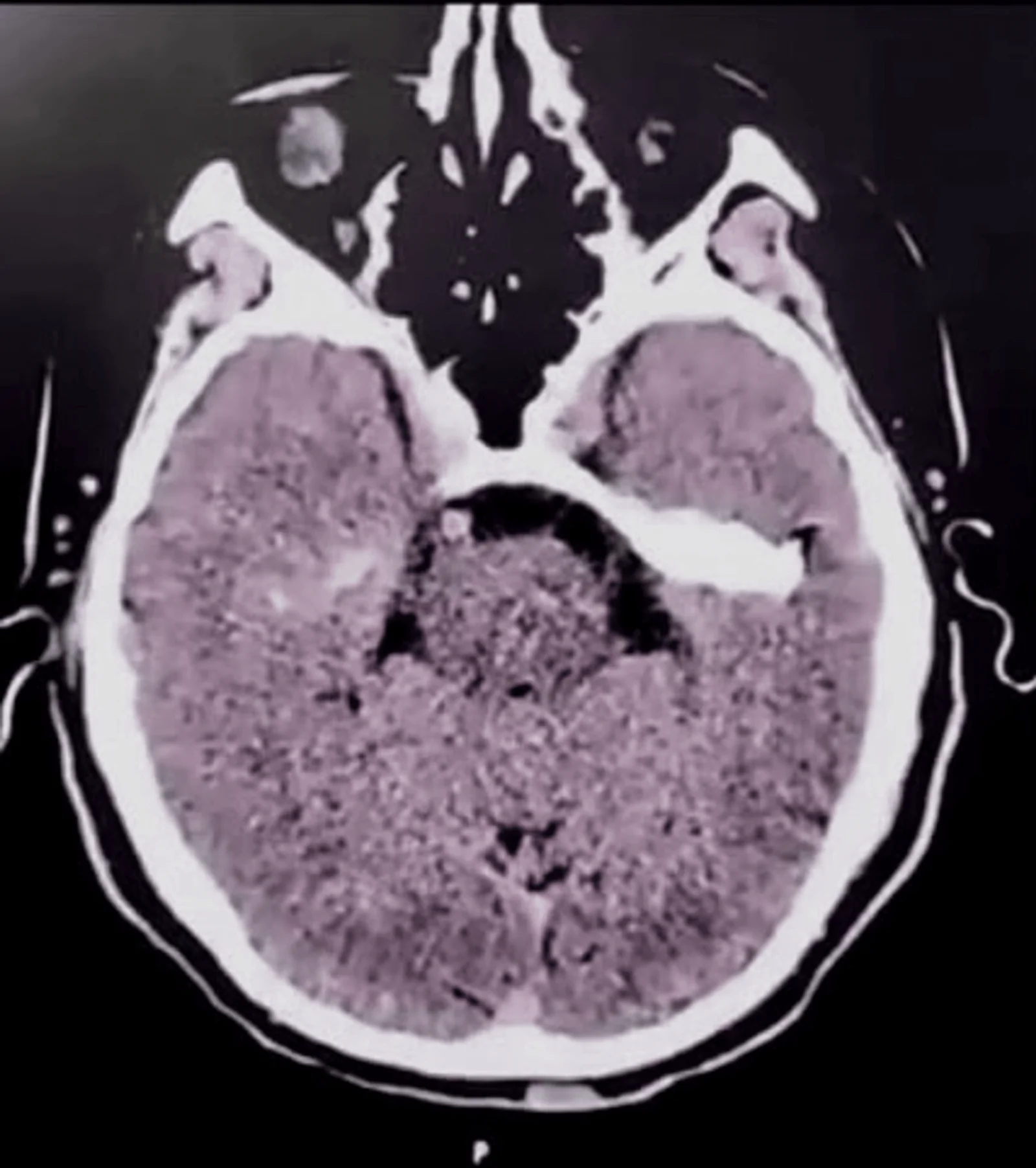
Axial non-contrast CT of the head at the level of the posterior fossa and basal cisterns.

Initial laboratory findings revealed leukocytosis (17.6x10³/μL; reference range: 4.0-11.0x10³/μL), with no infectious cause identified. A simple brain computed tomography (CT) scan was performed without any structural abnormalities or ischemic or hemorrhagic lesions; he was admitted to the internal medicine ward to evaluate the case further (Figures 1, 2).

During the hospital stay, a lumbar puncture was performed; findings included elevated protein levels (69.14 mg/dL; reference range 15-45 mg/dL) without pleocytosis or evidence suggesting a neurological infection. A meningitis/encephalitis panel was also performed; it included testing for *Listeria monocytogenes, Streptococcus pneumoniae, Streptococcus agalactiae, Escherichia coli, Haemophilus influenzae, Neisseria meningitidis, Cryptococcus neoformans/gattii*, human parechovirus, enterovirus, cytomegalovirus, human herpesvirus 6, varicella-zoster virus, and herpes simplex virus 1 and 2 (Table 1). The negative results across this panel helped rule out the most common bacterial, viral, and fungal causes of meningitis and encephalitis, supporting the exclusion of an infectious etiology for the patient's neurological presentation and reinforcing the diagnosis of GBS. Electrophysiological studies were not performed due to their unavailability at our institution, and anti-ganglioside antibodies were not requested for the same reason. Brighton diagnostic criteria could not be fully applied to the case due to the absence of electrophysiological studies and the atypical clinical manifestations; only four of the seven criteria could be evaluated. Regarding the differential diagnosis of bilateral facial palsy, idiopathic bilateral Bell's palsy was considered, whereas Melkersson-Rosenthal syndrome and carcinomatous meningitis were not formally considered given the patient's clinical presentation and course. The findings were compatible with atypical Guillain-Barré syndrome, and intravenous immunoglobulin treatment was initiated for five days.

**Table 1:** Meningitis/encephalitis panel results. Panel performed using a multiplex PCR assay.

Pathogen	Result	Reference value
Listeria monocytogenes	Not detected	Not detected
Streptococcus pneumoniae	Not detected	Not detected
Human parechovirus	Not detected	Not detected
Varicella-zoster virus	Not detected	Not detected
Cryptococcus neoformans/gattii	Not detected	Not detected
*Escherichia coli* KI	Not detected	Not detected
Streptococcus agalactiae	Not detected	Not detected
Haemophilus influenzae	Not detected	Not detected
Human herpesvirus 6	Not detected	Not detected
Enterovirus	Not detected	Not detected
Neisseria meningitidis	Not detected	Not detected
Cytomegalovirus	Not detected	Not detected
Herpes simplex virus 1	Not detected	Not detected
Herpes simplex virus 2	Not detected	Not detected

The patient experienced a favorable clinical course, with progressive improvement of dysarthria and facial weakness. There was no respiratory compromise, which was assessed with serial arterial blood gases and the absence of clinical signs of respiratory distress. There were no autonomic alterations and no further neurological deterioration. The patient was discharged successfully and without other medical complications. Regarding follow-up, the patient was referred to a third-level center for continued care after stabilization; as our institution lacks a formal mechanism to track outcomes of referred patients, we are unable to report follow-up data beyond the index hospitalization.

## Discussion

Guillain-Barré syndrome is an inflammatory disease of the peripheral nervous system with a heterogeneous presentation, including variants such as pure motor forms, bilateral facial palsy with paresthesias, pharyngeal-cervical-brachial weakness, a paraparetic presentation, and Miller-Fisher syndrome. These variants rarely present in isolated form because they usually share clinical features with the classical presentation or exhibit features of other well-described variants.

The pathophysiology is classically explained by an autoimmune process following an infection, with the most common viral triggers being cytomegalovirus and Epstein-Barr virus, and the most common bacterial trigger being *Campylobacter jejuni*. Recent studies have demonstrated an association between GBS and vaccination, including seasonal flu, hepatitis B, and human papillomavirus (Gardasil) [3].

The diagnosis of GBS is based on clinical findings and past medical history, supported by findings in complementary testing. The National Institute of Neurological Disorders and Stroke (NINDS) diagnostic criteria may help diagnose typical and atypical forms. The measurement of anti-ganglioside antibodies may prove useful when there is doubt in the diagnosis; however, a negative result does not rule out the diagnosis. It is not recommended to wait for the results of this test to start treatment [4,5].

Treatment is not determined by serum antibody levels or by presence or absence of specific symptoms. The clinical context of the patient must be individualized and must take into account the possibility of rapid progression of muscle weakness, autonomic dysfunction, bulbar compromise, or respiratory insufficiency. The first choice of treatment is intravenous immunoglobulin at 0.4 g/kg body weight over five days, or plasmapheresis for five days. Our patient presented with clinical features suggestive of GBS with bulbar involvement, without radiologic findings suggesting a structural or vascular origin. IV immunoglobulin was administered for five days, with an adequate clinical response.

## Conclusions

The clinical variants of GBS make this diagnosis a challenge, with bilateral facial palsy with paresthesias accounting for less than 1% of cases. This case is relevant because of the atypical features of bilateral facial diplegia, preceded by an episode of diarrhea - the most extensively studied predisposing factor for GBS - and, to a lesser extent, recent influenza vaccination, which cannot be entirely excluded as a contributing or even causal factor. Although classic sensorimotor involvement was absent, bilateral facial palsy with patchy paresthesias was present, and cerebrospinal fluid analysis and the exclusion of alternative diagnoses supported the final diagnosis. Electromyography and anti-ganglioside antibody testing, although part of the standard diagnostic workup for this variant, were not performed in this case; nonetheless, their absence does not rule out the diagnosis, which can be established on the basis of clinical features and CSF findings alone. This case reinforces the importance of maintaining a high index of suspicion for GBS even in the absence of typical symptoms, and the early use of immunotherapy once GBS is clinically suspected.

## References

[1] Habib AA, Waheed W (2023). Guillain-Barré syndrome. Continuum (Minneap Minn).

[2] Kumar P, Charaniya R, Bahl A, Ghosh A, Dixit J (2016). Facial diplegia with paresthesia: an uncommon variant of Guillain-Barré syndrome. J Clin Diagn Res.

[3] Shaikh AG, Termsarasab P, Nwankwo C, Rao-Frisch A, Katirji B (2012). Atypical forms of Guillain-Barré syndrome and H1N1-influenza vaccination. Vaccine.

[4] Leonhard SE, Mandarakas MR, Gondim FA (2019). Diagnosis and management of Guillain-Barré syndrome in ten steps. Nat Rev Neurol.

[5] Xu X, Wang Z, Su C, Cong L, Zheng D (2024). Case report: bilateral facial palsy with paresthesias and positive anti-GT1a antibodies. Front Immunol.

